# The Effectiveness of Early Food Introduction in Preventing Childhood Allergic Diseases: Protocol for a Systematic Review and Meta-Analysis

**DOI:** 10.2196/46816

**Published:** 2023-06-26

**Authors:** Aisha Fadhilah Abang Abdullah, Nor Asiah Muhamad, Rimah Melati Ab Ghani, Nur Hasnah Maamor, Fatin Norhasny Leman, Chun Lai Too, Intan Hakimah Ismail, Nor Afiah Mohd Zulkefli, Ahmad Iqmer Nashriq Mohd Nazan, Salmiah Md Said

**Affiliations:** 1 Department of Community Health Faculty of Medicine and Health Sciences Universiti Putra Malaysia Selangor Malaysia; 2 Department of Paediatrics Faculty of Medicine and Health Sciences Universiti Putra Malaysia Selangor Malaysia; 3 Sector for Evidence-based Healthcare National Institutes of Health Ministry of Health Selangor Malaysia; 4 Immunogenetic Unit, Allergy and Immunology Research Center Institute for Medical Research, National Institutes of Health Ministry of Health Shah Alam Malaysia

**Keywords:** protocol, systematic review, childhood allergic disease, weaning, early food introduction, food allergy, allergy, anaphylaxis, randomized controlled trial, pediatric, infant, childhood

## Abstract

**Background:**

Allergic diseases affect around 40% of the pediatric population worldwide. The coexistence of asthma, allergic rhinitis, eczema, and food allergy renders allergy treatment and prevention challenging. Infant feeding strategies recommend avoiding allergenic foods to prevent allergy development and anaphylaxis. However, recent evidence suggests that early consumption of food allergens during weaning in infants aged 4-6 months could result in food tolerance, thus reducing the risk of developing allergies.

**Objective:**

The aim of this study is to systematically review and carry out a meta-analysis of evidence on the outcome of early food introduction for preventing childhood allergic diseases.

**Methods:**

We will conduct a systematic review of interventions through a comprehensive search of various databases including PubMed, Embase, Scopus, CENTRAL, PsycINFO, CINAHL, and Google Scholar to identify potential studies. The search will be performed for any eligible articles from the earliest published articles up to the latest available studies in 2023. We will include randomized controlled trials (RCTs), cluster RCTs, non-RCTs, and other observational studies that assess the effect of early food introduction to prevent childhood allergic diseases.

**Results:**

Primary outcomes will include measures related to the effect of childhood allergic diseases (ie, asthma, allergic rhinitis, eczema, and food allergy). PRISMA (Preferred Reporting Items for Systematic Reviews and Meta-Analyses) guidelines will be followed for study selection. All data will be extracted using a standardized data extraction form and the quality of the studies will be assessed using the Cochrane Risk of Bias tool. A *summary of findings* table will be generated for the following outcomes: (1) total number of allergic diseases, (2) rate of sensitization, (3) total number of adverse events, (4) improvement of health-related quality of life, and (5) all-cause mortality. Descriptive and meta-analyses will be performed using a random-effects model in Review Manager (Cochrane). Heterogeneity among selected studies will be assessed using the *I*^2^ statistic and explored through meta-regression and subgroup analyses. Data collection is expected to start in June 2023.

**Conclusions:**

The results acquired from this study will contribute to the existing literature and harmonize recommendations for infant feeding with regard to the prevention of childhood allergic diseases.

**Trial Registration:**

PROSPERO CRD42021256776; https://tinyurl.com/4j272y8a

**International Registered Report Identifier (IRRID):**

PRR1-10.2196/46816

## Introduction

### Background

There has been a steep increase in the reported prevalence of common childhood allergic diseases (namely asthma, allergic rhinitis, eczema, and food allergy) in the last few decades [1-3]. According to Asher and Pearce [1], the International Study of Asthma and Allergies in Childhood concluded in the early 2000s reported that the prevalence of asthma, allergic rhinitis, and eczema had risen to over 30% in most industrialized countries. In addition, asthma is prevalent among approximately 300 million people worldwide, and by 2025, a further 100 million will likely be affected [4]. It is therefore crucial to evaluate the effectiveness of early life prevention techniques in order to lower the burden of childhood allergic diseases.

Infant feeding guidelines recommend early initiation of breastfeeding, including exclusive breastfeeding for the first 6 months of life and continued breastfeeding up to and after 2 years of age. Additionally, weaning or complementary feeding—defined as the provision of nutrition in the form of liquid or solid other than breast milk or infant milk formula—is introduced to neonates by 6 months of age, depending on the achievement of developmental milestones and the availability of safe complementary foods [5,6]*.* Besides providing essential nutrients to the growing infants, weaning exposes the developing gut microbiome to different antigens, thus influencing immune system development [7]. Food sensitization frequently occurs early in life and is often the first sign of future allergic disease; this is because the infant’s immune system has yet to develop, if at all, an allergic phenotype [8]. Food items with allergenic properties may resist digestion and enhance allergenicity. Hence, susceptible infants who did not develop tolerance will manifest an allergic phenotype. Additionally, the preservation of “beneficial” gut microbiota in the neonate prevents immunity alteration that subsequently predisposes him/her from developing an allergy [9]. As a result, many researchers propose nutritional allergy interventions during this period, such as prolonged breastfeeding, early introduction of foods, maternal avoidance diets during pregnancy and lactation, use of hypoallergenic formulas, and early allergen avoidance, albeit with varying degrees of success [8,10,11].

The landmark study, Learning Early About Peanut Allergy, was one of the first to suggest early introduction of peanuts to reduce the development of peanut allergies [12]. In 2016, the Enquiring about Tolerance study expanded to multiple allergenic food items (ie, peanuts, cooked eggs, cow’s milk, sesame, whitefish, and wheat) and observed a significant reduction in the prevalence of food allergies in the studied population [13]. These studies, along with others, appear to offer an effective and robust strategy to minimize the burden of allergy in the general population [10,14].

Living with allergic disease affects the quality of life of both patients and their guardians, considering the increasing health care costs. The determinants of health-related quality of life (HRQoL) in children with allergies traverse beyond biophysiological parameters and the symptom status of the child (such as physical limitations and emotional stress) and may include family dynamics and socioeconomic status, the community in which they live (ie, restrictions on participation in activities and social interaction), and organizational and policy-related factors (eg, inadequate food labelling and public information on allergies) [15-17]. Thus, effective allergy management strategy, including allergy prevention is imperative to avert allergic episodes that can be life-threatening.

Current recommendations for infant feeding require more consensus in the area of early life nutrition, specifically for prevention of childhood allergic diseases [18]. Moreover, an increasing number of studies have been examining the effect of early food introduction on reducing allergic sensitization, thus promoting allergy tolerance in these children [19-21]. We acknowledge earlier reports; however, most reviews have focused on the development of food allergies as well as having a diverse methodological approach [22-25]. In addition, there are diverse dietary patterns that may influence the development and progression of allergic diseases and their phenotypic expression in Asian populations. Although the published studies might have promising findings, less is understood about the potential harm that might result from the population-based guidelines and public health programs [26]. Thus, the aim of this systematic review is to identify evidence regarding the effect of early food introduction in preventing childhood allergic diseases.

### Objective

This study aims to systematically review and carry out a meta-analysis of the effectiveness of early food introduction in preventing childhood allergic diseases.

## Methods

This systematic review and meta-analysis will be conducted in accordance with the PRISMA (Preferred Reporting Items for Systematic Reviews and Meta-Analyses) statement [27] (Multimedia Appendix 1) and the Cochrane Handbook for Systematic Reviews of Interventions [28].

### Study Population and Selection Criteria

#### Inclusion Criteria

Our inclusion criteria will be based on the PICOS framework where the population of interest (“P”) is defined as comprising healthy infants who were introduced to early feeding, the intervention (“I”) is early introduction of complementary food (allergenic and nonallergenic) as a method of weaning when an infant approaches 4 months of age (ie, before turning 6 months old)—complementary food introduction is defined by the provision of nutrition other than breast milk or infants’ milk formula [29]. Weaning may involve liquid food, as in formula feeding, or solid food that provides essential nutrients to an infant and the developing gut microbiome while influencing immune development [30]. We will accept other interventions that were not specified in this study and were defined by individual authors if they do not involve any pharmacological agent. We will compare (“C”) our study cohort with infants who have been initiated on complementary feeding (ie, weaning) in accordance with the standard infant feeding guidelines as described by the studies included in this meta-analysis. The World Health Organization and United Nations International Children's Emergency Fund recommend early initiation of breastfeeding, including exclusive breastfeeding for the first 6 months of life followed by continued breastfeeding for up to 2 years and beyond [31]. Additionally, adequate complementary foods are recommended from the beginning of 4 months to latest 6 months of age to all neonates [32]. The outcomes (“O”) are categorized as (1) primary outcomes including the total number of cases presented with asthma, allergic rhinitis, eczema, and food allergy (a reproducible hypersensitivity reaction to certain food items) and (2) secondary outcomes such as allergic sensitization (ie, the presence of elevated total and specific immunoglobulin E (IgE) levels to an allergen [33]) and total number of adverse events from allergen exposure. We will also evaluate studies reporting improvement in HRQoL, measured using validated scales (eg, Pediatric Allergic Disease Quality of Life Questionnaire, Food Allergy Quality of Life Questionnaires, and Pediatric Quality of Life Inventory) as well as all-cause mortality. We will include studies (“S”) that are randomized controlled trials (RCTs) or cluster-RCTs, non-RCTs, and other observational studies that evaluate the effectiveness of early food introduction for preventing childhood allergic diseases. All published reports from inception up to October 2023 will be included.

#### Exclusion Criteria

Excluded studies are those that enrolled infants older than 6 months, those that involved food interventions among infants with congenital abnormalities (eg, dysmorphism), those not published in English, all unpublished trials and abstracts, and those describing multicomponent management (studies including the use of drugs). We will also exclude studies where infants initiated on supplementary feeding before the age of 4 months and those in which infants were initiated on certain immune suppressors (eg, steroids). We will exclude cross-over studies due to concerns about the carryover effects. We will also exclude all observational studies, any short communication, case report, and guidelines. Table 1 summarizes the PICOS search strategies, and sources of review for this study.

**Table 1:** PICO^a^ search strategies and sources of review.

	Sources based on the PRISMA^b^ checklist
Population	Healthy infants at recruitment as outlined by study authors.
Intervention	Early food introduction or early weaning when an infant approaches 4 months of age and before turning 6 months old.
Comparison	Infants initiated on complementary feeding (weaning) in accordance with standard infant feeding guidelines as described by the author.
Outcome	Primary outcome: total number of events of allergic disease (including asthma, allergic rhinitis, eczema, and food allergy). Secondary outcomes: rate of sensitization (level of total and specific immunoglobulin E), total number of adverse events, improvement of health-related quality of life measured using validated scales, and all-cause mortality.

^a^PICOS: population, intervention, compare, outcomes, and studies.

^b^PRISMA: Preferred Reporting Items for Systematic Reviews and Meta-Analyses.

### Search Strategy

A systematic and comprehensive literature search will be conducted in various databases including PubMed, CENTRAL, Embase, Scopus, PsycINFO, CINAHL, and Google Scholar from inception to October 2023. A search strategy will be developed for PubMed and adapted for use with other electronic databases (Multimedia Appendix 2). The search will include MeSH terms and keywords relating to the study population (“childhood” OR “children” OR “paediatric” OR “pediatrics” OR “infants”), intervention (“early food introduction” OR “weaning” OR “complementary feeding” OR “supplementary feeding”), and outcomes (“allergies” OR “allergy” OR “allergic diseases” OR “asthma” OR “allergic rhinitis” OR “eczema” OR “dermatitis” OR “food allergy” OR “sensitization”).

### Data Collection

Standard Cochrane methods will be used, as described in the Cochrane Handbook for Systematic Reviews of Interventions [34]. Two authors will independently screen all the titles and abstracts to examine all the potential studies. We will code them as “retrieve” (eligible, or potentially eligible or unclear) or “do not retrieve.” We will retrieve full-text reports or publications, and 2 other review authors will independently screen the full text to identify studies for inclusion and will identify and record the reasons for exclusion of the ineligible studies. We will use a standardized data extraction form in accordance with the Cochrane Handbook for Systematic Reviews of Intervention [28]. Data will be independently extracted in accordance with general information (author, source, date of publication, country, and language), study (type of study, study duration, sample size, and control group selection), participants (age, sex, and type of allergy), types of intervention (food avoidance upon diagnosis and food challenge), and, lastly, outcomes (allergic episodes, admissions or emergency visits, duration of illness, and quality of life measured using validated scales) [28].

**Figure 1:**
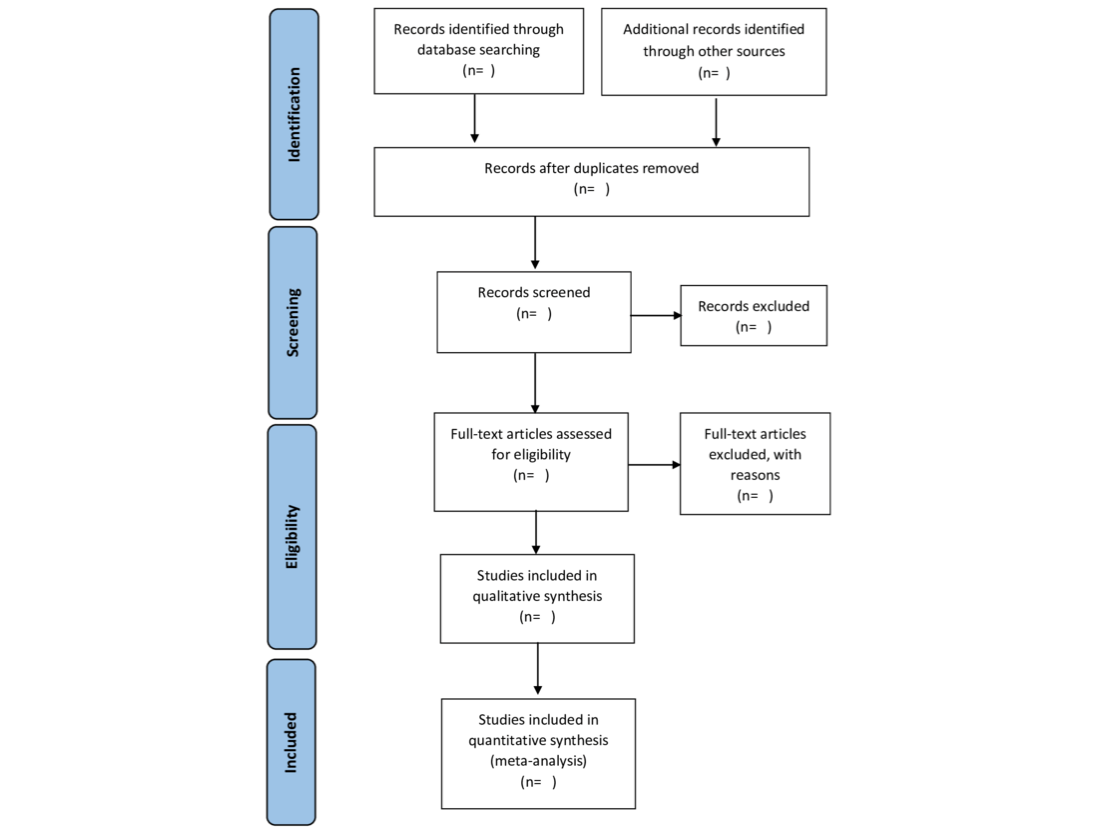
The PRISMA (Preferred Reporting Items for Systematic Reviews and Meta-Analyses) flowchart for reporting studies.

We will resolve any disagreement through discussion or consult a third review author if necessary. We will identify and exclude duplicates, and if multiple or overlapped reports of the same study are found, we will group them under a single study ID, and assign the report with the most amount of relevant information as the primary publication. We will record the selection process in sufficient detail to complete a PRISMA flow diagram (Figure 1) and construct a table describing the characteristics of the excluded studies [35]. We will resolve any discrepancies through discussion and by involving a third author.

### Quality Assessment of the Included Studies

Two reviewers will independently assess the quality of the included studies. The methodological quality of selected studies will be assessed using the Cochrane Risk of Bias Tool [36] for RCTs and cluster-RCTs, which includes the following domains: allocation concealment of random sequence generation, blinding of participants and personnel, blinding of outcome assessment, incomplete outcome data, selective outcome reporting, and other bias. We will use ACROBAT-NRSi (A Cochrane Risk Of Bias Assessment Tool for Non-Randomized Studies), which evaluates the risk of bias in the results of non-RCTs studies that compare the health effects of 2 or more interventions [37]. We will use Newcastle-Ottawa scale for observational studies and evaluate the quality parameters (selection, comparability, and outcome) of the selected studies [38]. We will make judgments on each of the abovementioned criteria regarding whether the study has a high, low, or unclear risk of bias. We will summarize the risk-of-bias judgements with justification for each of the domains listed in the *Risk of bias* table. Any disagreement among the review authors will be resolved by discussion to achieve a consensus.

### Summary of Findings Table

We will create a *summary of findings* table by tabulating the following outcomes: (1) total number of allergic diseases (asthma, allergic rhinitis, eczema, and food allergy), (2) rate of sensitization (level of total and specific IgE), (3) total number of adverse events, (4) improvement of HRQoL measured using validated questionnaires, and (5) all-cause mortality [39]. We will use the following 5 GRADE (Grading of Recommendations Assessment, Development and Evaluation) elements to assess the quality of the body of evidence as it relates to the prespecified outcomes: quality of evidence, consistency of effect, imprecision, indirectness, and publication bias. We will assess the quality of evidence for all outcomes using the GRADE methodology [40].

### Data Analysis

#### Statistical Analysis

A meta-analysis will be conducted if there are at least 2 similar studies with broadly similar populations, interventions, and outcome measures using a random-effects model in Review Manager (Cochrane) [41]. A random-effects model will be used if studies are statistically heterogeneous; otherwise, we will use a fixed-effects model. For the random-effects model, we will conduct a sensitivity check by using the fixed-effects model to reveal differences in the results. We will include a 95% CI for all estimates. We will report skewed data as medians and IQRs. Studies will be considered as being sufficiently similar if the relevant outcome data are available in the group originally allocated or if the intervention, such as early food introduction in the allocated group, belongs to the same category—for example, the same amount of food (eg, frequency or total daily intake)—and if only frequencies are reported, we will include binary comparisons; for example, weekly versus never and daily versus never. The primary data analysis will include proportions or the frequency of childhood allergic diseases and types of food (allergenic and nonallergenic).

#### Assessment of Heterogeneity

The clinical heterogeneity of the results of the included studies will be assessed on the basis of the similarity of their populations, interventions, outcomes, and follow-up if we can pool sufficient studies together. We will consider populations as similar when they are in the same categories of age with outcomes measured in similar ways as stated in the eligibility criteria. We will consider interventions as similar if they fall into the same category as indicated in the abovementioned types of interventions. The outcomes will be considered as similar when they are measured at similar events as stated in the eligibility criteria. We will estimate the treatment effects of individual RCTs and examine heterogeneity among RCTs by inspecting forest plots and quantifying the impact of heterogeneity using the *I*^2^ statistic [42].

#### Assessment of Reporting Biases

We will generate and examine a funnel plot to explore possible small biases in studies if we can pool more than 5 RCTs in a single meta-analysis.

### Ethics and Dissemination

This systematic review is registered with the National Medical Research Register, Ministry of Health Malaysia (ID: NMRR ID-22-01049-HQ4). This protocol registered in PROSPERO (CRD42021256776). We will present the findings of this review in National and international conferences and continuous medical education initiatives, and we will disseminate our findings to policy makers and top managements (stakeholders) of Ministry of Health Malaysia.

## Results

The protocol has been registered in PROSPERO and we initiated the review on June 1, 2023, and the results are expected by October 1, 2023. This systematic review and meta-analysis shall contribute an update to the existing literature on infant feeding with emphasis on the risk of childhood allergic diseases.

## Discussion

### Anticipated Findings

**Figure 2:**
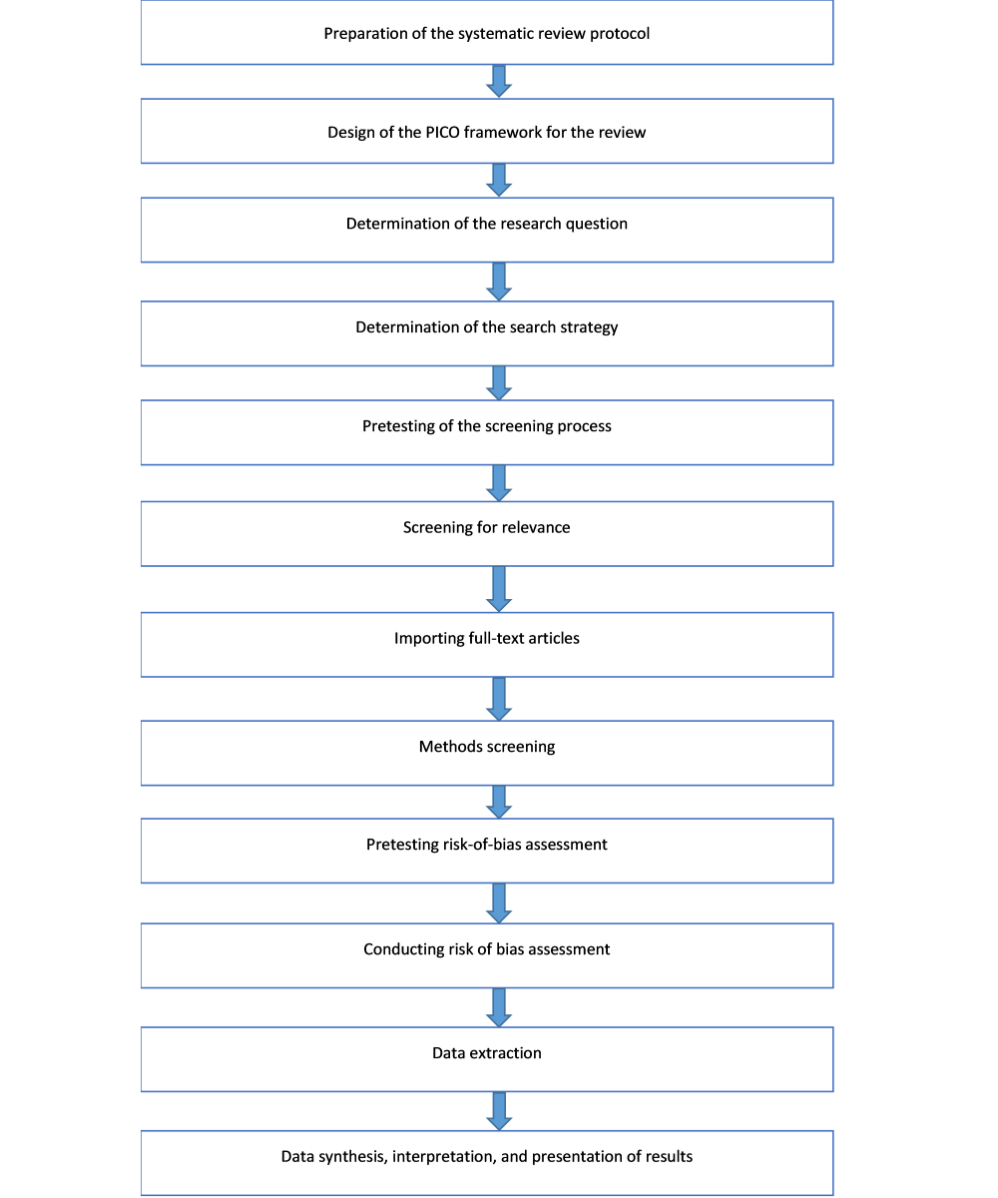
Flow diagram of the study process. PICOS: population, intervention, compare, outcomes, and studies.

This protocol outlines the methods for a systematic review of the literature to assess the effectiveness of early food introduction in the prevention of childhood allergic diseases. Figure 2 illustrates a flow diagram of the study process to be followed as a guide for this review in terms of literature search using keywords identified through discussion among authors, the screening of shortlisted studies following a standardized framework, and the process of full text retrieval. This will be followed by quality assessment with data extraction for evidence synthesis and finally publishing of the study results.

We take note of 2 similar systematic reviews and meta-analyses that are closely related to our study [23,43]. However, their methodology (in terms of population variability and age of weaning) and diverse outcomes are different from those based on our study objective. In our study, we aim specifically to evaluate the effectiveness of early food introduction in infants aged 4-6 months irrespective of risks, and to evaluate outcomes related to 4 common allergic diseases in children. We believe that our findings will provide a reasonable and robust update to the existing literature.

### Reaching Conclusions

We will base our conclusions only on findings from the quantitative or narrative synthesis of the studies included in this review. The conclusions can be used as guidelines for top managers and stakeholders for evidence-informed policy on infant feeding guidelines for the prevention of childhood allergic diseases. Our implications for research will suggest priorities for future research and outline the remaining uncertainties in the area.

## Appendix

Multimedia Appendix 1 PRISMA-P (Preferred Reporting Items for Systematic Reviews and Meta-Analyses Protocols) checklist.

Multimedia Appendix 2 Search strategy developed for PubMed.

## Abbreviations

ACROBAT-NRSi: A Cochrane Risk Of Bias Assessment Tool for Non-Randomized Studies
GRADE: Grading of Recommendations Assessment, Development and Evaluation
HRQoL: health-related quality of life
IgE: immunoglobulin E
PICOS: population, intervention, compare, outcomes, and studies
PRISMA: Preferred Reporting Items for Systematic Reviews and Meta-Analyses
RCT: randomized controlled trial

## Data Availability

The original contributions shown in the study are contained within this systematic review and its multimedia appendices; further inquiries can be addressed to the corresponding author.
