# Supplementary material for: The Effectiveness of Early Food Introduction in Preventing Childhood Allergic Diseases: Protocol for a Systematic Review and Meta-Analysis
Source: JMIR Res Protoc. 2023 Jun 26;12:e46816. doi: 10.2196/46816 (PMC10337433; doi:10.2196/46816)
Supplement: Multimedia Appendix 2 [file resprot_v12i1e46816_app2.pdf]

Appendix1. Search strategy for PubMed

No	Search Strategy
1	healthy infan*[Title/Abstract]
2	healthy infan*[MeSH Terms]
3	healthy newborn[Title/Abstract]
4	healthy newborn[MeSH Terms]
5	healthy newborn infant[Title/Abstract]
6	healthy newborn infant[MeSH Terms]
7	healthy baby[Title/Abstract]
8	healthy baby[MeSH Terms]
9	healthy child*[Title/Abstract]
10	healthy child*[MeSH Terms]
11	healthy young child[Title/Abstract]
12	healthy young child[MeSH Terms]
13	healthy toddler*[Title/Abstract]
14	healthy neonat*[Title/Abstract]
15	healthy neonat*[MeSH Terms]
16	healthy pediatrics[Title/Abstract]
17	healthy pediatrics[MeSH Terms]
18	healthy paediatrics[Title/Abstract]
19	healthy paediatrics[MeSH Terms]
20	(((((((((((((((((((healthy infan*[Title/Abstract]) OR (healthy infan*[MeSH Terms])) OR (healthy newborn[Title/Abstract])) OR (healthy newborn[MeSH Terms])) OR (healthy newborn infant[Title/Abstract])) OR (healthy newborn infant[MeSH Terms])) OR (healthy baby[Title/Abstract]))) OR (healthy baby[MeSH Terms])) OR (healthy child*[Title/Abstract])) OR (healthy child*[MeSH Terms])) OR (healthy young child[Title/Abstract])) OR (healthy young child[MeSH Terms])) OR (healthy toddler*[Title/Abstract])) OR (healthy neonat*[Title/Abstract])) OR (healthy neonat*[MeSH Terms])) OR (healthy pediatrics[Title/Abstract])) OR (healthy pediatrics[MeSH Terms])) OR (healthy paediatrics[Title/Abstract])) OR (healthy paediatrics[MeSH Terms])
21	Wean*[Title/Abstract]
22	Wean*[MeSH Terms]
23	weaning food[Title/Abstract]
24	weaning food[MeSH Terms]
25	complementary food[Title/Abstract]
26	complementary food[MeSH Terms]
27	complementary feeding[Title/Abstract]
28	complementary feeding[MeSH Terms]
29	supplementary feeding[Title/Abstract]
30	supplementary feeding[MeSH Terms]
31	early food introduction[Title/Abstract]

32	solid food introduction[Title/Abstract]
33	introducing solid food[Title/Abstract]
34	introducing solid food[MeSH Terms]
35	early feeding food[Title/Abstract]
36	early feeding food[MeSH Terms]
37	first food[Title/Abstract]
38	first food[MeSH Terms]
39	(((((((((((((((((Wean*[Title/Abstract]) OR (Wean*[MeSH Terms])) OR (weaning food[Title/Abstract])) OR (weaning food[MeSH Terms])) OR (complementary food[Title/Abstract])) OR (complementary food[MeSH Terms])) OR (complementary feeding[Title/Abstract])) OR (complementary feeding[MeSH Terms])) OR (supplementary feeding[Title/Abstract])) OR (supplementary feeding[MeSH Terms])) OR (early food introduction[Title/Abstract])) OR (solid food introduction[Title/Abstract])) OR (introducing solid food[Title/Abstract])) OR (introducing solid food[MeSH Terms])) OR (early feeding food[Title/Abstract])) OR (early feeding food[MeSH Terms])) OR (first food[Title/Abstract])) OR (first food[MeSH Terms])
40	Standard wean* food[Title/Abstract]
41	Standard wean* food[MeSH Terms]
42	(Standard wean* food[Title/Abstract]) OR (Standard wean* food[MeSH Terms])
43	Incidence allergic diseases[Title/Abstract]
44	Incidence allergic diseases[MeSH Terms]
45	Incidence eczema[Title/Abstract]
46	Incidence eczema[MeSH Terms]
47	Incidence allergic rhinitis[Title/Abstract]
48	Incidence asthma[Title/Abstract]
49	Incidence asthma[MeSH Terms]
50	Incidence food allergy[Title/Abstract]
51	Incidence food allergy[MeSH Terms]
52	Number allergic diseases[Title/Abstract]
53	Number allergic diseases[MeSH Terms]
54	Number eczema[Title/Abstract]
55	Number eczema[MeSH Terms]
56	Number allergic rhinitis[Title/Abstract]
57	Number allergic rhinitis[MeSH Terms]
58	Number asthma[Title/Abstract]
59	Number asthma[MeSH Terms]
60	Number food allergy[Title/Abstract]
61	Number food allergy[MeSH Terms]
62	Sensitization[Title/Abstract]
63	Immunologic* sensitization[Title/Abstract]
64	Immunoglobulin E[Title/Abstract]
65	Immunoglobulin E[MeSH Terms]

66	IgE[Title/Abstract]
67	cause mortality[Title/Abstract]
68	cause mortality[MeSH Terms]
69	mortality[Title/Abstract]
70	mortality[MeSH Terms]
71	death[Title/Abstract]
72	death[MeSH Terms]
73	adverse events[Title/Abstract]
74	anaphylaxis[Title/Abstract]
75	anaphylaxis[MeSH Terms]
76	urticaria[Title/Abstract]
77	urticaria[MeSH Terms]
78	vomiting[Title/Abstract]
79	vomiting[MeSH Terms]
80	allergic rashes[Title/Abstract]
81	allergic rashes[MeSH Terms]
82	hypotension[Title/Abstract]
83	(hypotension[MeSH Terms]) AND (hypotension[MeSH Terms])
84	shortness of breath[Title/Abstract]
85	shortness of breath[MeSH Terms]
86	difficulty in breathing[Title/Abstract]
87	difficulty in breathing[MeSH Terms]
88	wheezing[Title/Abstract]
89	wheezing[MeSH Terms]
90	syncope[Title/Abstract]
91	syncope[MeSH Terms]
92	Malnutrition[Title/Abstract]
93	Malnutrition[MeSH Terms]
94	Stunted growth[Title/Abstract]
95	Stunted growth[MeSH Terms]
96	HRQoL[Title/Abstract]
97	HRQoL[MeSH Terms]
98	health-related quality of life[Title/Abstract]
99	health-related quality of life[MeSH Terms]
100	((Incidence allergic diseases[Title/Abstract]) OR (Incidence eczema[Title/Abstract])) OR (Incidence eczema[MeSH Terms])) OR (Incidence allergic rhinitis[Title/Abstract])) OR (Incidence asthma[Title/Abstract])) OR (Incidence asthma[MeSH Terms])) OR (Incidence food allergy[Title/Abstract])) OR (Incidence food allergy[MeSH Terms])) OR (Number allergic diseases[Title/Abstract])) OR (Number allergic diseases[MeSH Terms])) OR (Number eczema[Title/Abstract])) OR (Number eczema[MeSH Terms])) OR (Number allergic rhinitis[Title/Abstract])) OR (Number allergic rhinitis[MeSH Terms])) OR (Number asthma[Title/Abstract])) OR (Number asthma[MeSH Terms])) OR

	(Number food allergy[Title/Abstract])) OR (Number food allergy[MeSH Terms])) OR (Sensitization[Title/Abstract])) OR (Immunologic* sensitization[Title/Abstract])) OR (Immunoglobulin E[Title/Abstract])) OR (Immunoglobulin E[MeSH Terms])) OR (IgE[Title/Abstract])) OR (cause mortality[Title/Abstract])) OR (cause mortality[MeSH Terms])) OR (mortality[Title/Abstract])) OR (mortality[MeSH Terms])) OR (death[Title/Abstract])) OR (death[MeSH Terms])) OR (adverse events[Title/Abstract])) OR (anaphylaxis[Title/Abstract])) OR (anaphylaxis[MeSH Terms])) OR (urticaria[Title/Abstract])) OR (urticaria[MeSH Terms])) OR (vomiting[Title/Abstract])) OR (vomiting[MeSH Terms])) OR (allergic rashes[Title/Abstract])) OR (allergic rashes[MeSH Terms])) OR (hypotension[Title/Abstract])) OR ((hypotension[MeSH Terms]) AND (hypotension[MeSH Terms]))) OR (shortness of breath[Title/Abstract])) OR (shortness of breath[MeSH Terms])) OR (difficulty in breathing[Title/Abstract])) OR (difficulty in breathing[MeSH Terms])) OR (wheezing[Title/Abstract])) OR (wheezing[MeSH Terms])) OR (syncope[Title/Abstract])) OR (syncope[MeSH Terms])) OR (Malnutrition[Title/Abstract])) OR (Malnutrition[MeSH Terms])) OR (Stunted growth[Title/Abstract])) OR (Stunted growth[MeSH Terms])) OR (HRQoL[Title/Abstract])) OR (HRQoL[MeSH Terms])) OR (health-related quality of life[Title/Abstract])) OR (health-related quality of life[MeSH Terms])
101	(((((((((((((((((((((healthy infan*[Title/Abstract]) OR (healthy infan*[MeSH Terms])) OR (healthy newborn[Title/Abstract])) OR (healthy newborn[MeSH Terms])) OR (healthy newborn infant[Title/Abstract])) OR (healthy newborn infant[MeSH Terms])) OR (healthy baby[Title/Abstract]))) OR (healthy baby[MeSH Terms])) OR (healthy child*[Title/Abstract])) OR (healthy child*[MeSH Terms])) OR (healthy young child[Title/Abstract])) OR (healthy young child[MeSH Terms])) OR (healthy toddler*[Title/Abstract])) OR (healthy neonat*[Title/Abstract])) OR (healthy neonat*[MeSH Terms])) OR (healthy pediatrics[Title/Abstract])) OR (healthy pediatrics[MeSH Terms])) OR (healthy paediatrics[Title/Abstract])) OR (healthy paediatrics[MeSH Terms])) AND ((((((((((((((((((Wean*[Title/Abstract]) OR (Wean*[MeSH Terms])) OR (weaning food[Title/Abstract])) OR (weaning food[MeSH Terms])) OR (complementary food[Title/Abstract])) OR (complementary food[MeSH Terms])) OR (complementary feeding[Title/Abstract])) OR (complementary feeding[MeSH Terms])) OR (supplementary feeding[Title/Abstract])) OR (supplementary feeding[MeSH Terms])) OR (early food introduction[Title/Abstract])) OR (solid food introduction[Title/Abstract])) OR (introducing solid food[Title/Abstract])) OR (introducing solid food[MeSH Terms])) OR (early feeding food[Title/Abstract])) OR (early feeding food[MeSH Terms])) OR (first food[Title/Abstract])) OR (first food[MeSH Terms]))) AND ((Standard wean* food[Title/Abstract]) OR (Standard wean* food[MeSH Terms]))) AND ((Incidence allergic diseases[Title/Abstract]) OR (Incidence eczema[Title/Abstract])) OR (Incidence eczema[MeSH Terms])) OR (Incidence allergic rhinitis[Title/Abstract])) OR (Incidence asthma[Title/Abstract])) OR (Incidence asthma[MeSH Terms])) OR (Incidence food allergy[Title/Abstract])) OR (Incidence food allergy[MeSH Terms])) OR (Number allergic diseases[Title/Abstract])) OR (Number allergic diseases[MeSH Terms])) OR (Number eczema[Title/Abstract])) OR (Number eczema[MeSH Terms])) OR (Number allergic rhinitis[Title/Abstract])) OR (Number allergic rhinitis[MeSH Terms])) OR (Number asthma[Title/Abstract])) OR (Number asthma[MeSH Terms])) OR (Number food allergy[Title/Abstract])) OR (Number food allergy[MeSH Terms])) OR (Sensitization[Title/Abstract])) OR (Immunologic* sensitization[Title/Abstract])) OR (Immunoglobulin E[Title/Abstract])) OR (Immunoglobulin E[MeSH Terms])) OR

	(IgE[Title/Abstract])) OR (cause mortality[Title/Abstract])) OR (cause mortality[MeSH Terms])) OR (mortality[Title/Abstract])) OR (mortality[MeSH Terms])) OR (death[Title/Abstract])) OR (death[MeSH Terms])) OR (adverse events[Title/Abstract])) OR (anaphylaxis[Title/Abstract])) OR (anaphylaxis[MeSH Terms])) OR (urticaria[Title/Abstract])) OR (urticaria[MeSH Terms])) OR (vomiting[Title/Abstract])) OR (vomiting[MeSH Terms])) OR (allergic rashes[Title/Abstract])) OR (allergic rashes[MeSH Terms])) OR (hypotension[Title/Abstract])) OR ((hypotension[MeSH Terms]) AND (hypotension[MeSH Terms]))) OR (shortness of breath[Title/Abstract])) OR (shortness of breath[MeSH Terms])) OR (difficulty in breathing[Title/Abstract])) OR (difficulty in breathing[MeSH Terms])) OR (wheezing[Title/Abstract])) OR (wheezing[MeSH Terms])) OR (syncope[Title/Abstract])) OR (syncope[MeSH Terms])) OR (Malnutrition[Title/Abstract])) OR (Malnutrition[MeSH Terms])) OR (Stunted growth[Title/Abstract])) OR (Stunted growth[MeSH Terms])) OR (HRQoL[Title/Abstract])) OR (HRQoL[MeSH Terms])) OR (health-related quality of life[Title/Abstract])) OR (health-related quality of life[MeSH Terms]))
